# Point-of-care testing and treatment of sexually transmitted infections to improve birth outcomes in high-burden, low-income settings: Study protocol for a cluster randomized crossover trial (the WANTAIM Trial, Papua New Guinea)

**DOI:** 10.12688/wellcomeopenres.15173.2

**Published:** 2019-11-20

**Authors:** Andrew J. Vallely, William S. Pomat, Caroline Homer, Rebecca Guy, Stanley Luchters, Glen D. L. Mola, Grace Kariwiga, Lisa M. Vallely, Virginia Wiseman, Chris Morgan, Handan Wand, Stephen J. Rogerson, Sepehr N. Tabrizi, David M. Whiley, Nicola Low, Rosanna Peeling, Peter Siba, Michaela Riddell, Moses Laman, John Bolnga, Leanne J. Robinson, Jacob Morewaya, Steven G. Badman, Neha Batura, Angela Kelly-Hanku, Pamela J. Toliman, Wilfred Peter, Delly Babona, Elizabeth Peach, Suzanne M. Garland, John M. Kaldor

**Affiliations:** 1 Papua New Guinea Institute of Medical Research, Goroka, EHP, 441, Papua New Guinea; 2 The Kirby Institute for infection and immunity in society, UNSW Sydney, Sydney, NSW, 2052, Australia; 3 Macfarlane Burnet Institute for Medical Research and Public Health, Melbourne, VIC, 3004, Australia; 4 School of Medicine and Health Sciences, University of Papua New Guinea, Port Moresby, NCD, Papua New Guinea; 5 Milne Bay Provincial Health Authority, Alotau, MBP, Papua New Guinea; 6 London School of Hygiene & Tropical Medicine, London, WC1E 7HT, UK; 7 Doherty Institute, Department of Medicine, University of Melbourne, Melbourne, VIC, 3050, Australia; 8 Department of Microbiology, The Royal Women’s Hospital Melbourne, Parkville, VIC, 3052, Australia; 9 Department of Obstetrics and Gynaecology, University of Melbourne, Carlton, VIC, 3053, Australia; 10 UQ Centre for Clinical Research, University of Queensland, Herston, QLD, 4029, Australia; 11 Institute of Social and Preventive Medicine, University of Bern, Bern, 3012, Switzerland; 12 Department of Obstetrics & Gynaecology, Modilon General Hospital, Madang, MP, Papua New Guinea; 13 Centre for Global Health Economics, Institute for Global Health, University College London, London, WC1N 1EH, UK; 14 Provincial Health Office, Madang, MP, Papua New Guinea; 15 St Mary’s Vunapope Rural Hospital, Kokopo, ENBP, 613, Papua New Guinea

**Keywords:** sexually transmitted infection, pregnancy, preterm birth, low birth weight, cluster randomised crossover trial, Papua New Guinea

## Abstract

**Background:** *Chlamydia trachomatis*, *Neisseria gonorrhoeae*, *Trichomonas vaginalis* and bacterial vaginosis have been associated with preterm birth and low birth weight, and are highly prevalent among pregnant women in many low- and middle-income settings. There is conflicting evidence on the potential benefits of screening and treating these infections in pregnancy. Newly available diagnostic technologies make it possible, for the first time, to conduct definitive field trials to fill this knowledge gap. The primary aim of this study is to evaluate whether antenatal point-of-care testing and immediate treatment of these curable sexually transmitted and genital infections (STIs) leads to reduction in preterm birth and low birth weight.

**Methods**: The Women and Newborn Trial of Antenatal Interventions and Management (WANTAIM) is a cluster-randomised crossover trial in Papua New Guinea to compare point-of-care STI testing and immediate treatment with standard antenatal care (which includes the WHO-endorsed STI ‘syndromic’ management strategy based on clinical features alone without laboratory confirmation). The unit of randomisation is a primary health care facility and its catchment communities. The primary outcome is a composite measure of two events: the proportion of women and their newborns in each trial arm, who experience either preterm birth (delivery <37 completed weeks of gestation as determined by ultrasound) and/or low birth weight (<2500 g measured within 72 hours of birth). The trial will also evaluate neonatal outcomes, as well as the cost-effectiveness, acceptability and health system requirements of this strategy, compared with standard care.

**Conclusions: **WANTAIM is the first randomised trial to evaluate the effectiveness, cost-effectiveness, acceptability and health system requirements of point-of-care STI testing and treatment to improve birth outcomes in high-burden settings. If the intervention is proven to have an impact, the trial will hasten access to these technologies and could improve maternal and neonatal health in high-burden settings worldwide.

**Registration:** ISRCTN37134032.

## Introduction

### Background

Every year there are an estimated 2.6 million stillbirths and 2.6 million neonatal deaths globally; the majority are in low- and middle-income countries (LMIC), primarily in remote and rural communities ^1^. Papua New Guinea (PNG) has among the highest neonatal mortality ratios worldwide, with an estimated 25 per 1000 live births in 2013, compared with a global figure of 18 ^2, 3^. Preterm birth and low birth weight are closely linked, as well as being independent and major contributors to neonatal mortality. Together, they affect around 20% of newborns in PNG ^4^. The causes of preterm birth and low birth weight are diverse, but a range of infections including malaria, syphilis, and other sexually transmitted and genital infections (STI) have been implicated ^5– 10^. In many resource-limited countries, poor access to antenatal care means that opportunities for early diagnosis and clinical intervention of such infections are missed ^11^.

Research studies among antenatal women in a number of LMIC have found extremely high rates of infection with genital STIs, particularly gonorrhoea, chlamydia, trichomonas and bacterial vaginosis, which are readily curable with cheap antibiotics ^5^. In PNG, pregnant women have among the highest prevalence of these infections of any developing country ^12^. In the country’s first bio-behavioural survey of STIs in pregnancy, we found that the prevalence of chlamydia was 23%, gonorrhoea 14%, and trichomonas 22%, with 44% of women having at least one of these infections ^13^. Similar levels of infection were found in a pilot study of antenatal point-of-care STI testing and treatment conducted by our group ^14^ in which 54% of women had one or more of chlamydia, gonorrhoea, trichomonas or BV, and the prevalence of each of these STIs was 19%, 11%, 38%, and 18%, respectively. Similar STI prevalences were also observed in an earlier randomised trial of malaria prevention in pregnancy ^15^. In all of these studies, between 65–80% of infections among antenatal women were asymptomatic.

### STIs and adverse birth outcomes

Gonorrhoea, chlamydia, trichomonas and bacterial vaginosis have been linked to adverse birth outcomes. The precise pathogenesis remains unclear, with postulated mechanisms including direct infection of the foetus; stimulation of foetal inflammatory responses; excessive maternal immunogenic reactions; or a combination of factors ^16^. There is evidence that gonorrhoea and chlamydia are associated with both preterm birth and low birth weight ^5, 8, 9, 17– 23^, as well as miscarriage ^5, 21^, stillbirth ^5, 24^, premature rupture of membranes ^5, 17, 25^, postpartum endometritis ^5^ and ophthalmia neonatorum ^5^. The reported strength of association varies across studies and endpoints ^8, 26^. In a meta-analysis, we found that trichomonas is associated with a relative risk of 1.4 (95%CI: 1.1-1.7) for preterm birth ^27^, and is also linked to low birth weight and premature rupture of membranes. Bacterial vaginosis is strongly associated with preterm birth and other adverse outcomes ^17, 20, 28^. The population attributable risk of these infections as causes of adverse birth outcomes depends on their underlying population prevalence. For preterm birth, estimates have ranged from 15% for gonorrhoea, chlamydia, trichomonas and bacterial vaginosis individually, and up to 42% for a combination of one or more of these infections ^9, 19, 20^.

### Management of STIs in pregnancy

Diagnosis of genital infections has traditionally relied upon microscopy, culture, and/or serology, all of which require technical expertise and laboratory services which are not widely available in most resource-limited settings. Accordingly, in the 1990s, the World Health Organization (WHO) developed a syndromic management strategy for diagnosing genital infections that uses clinical presentation without laboratory confirmation to make treatment decisions. Syndromic management, however, cannot identify asymptomatic infections, which might contribute to its limited impact on disease prevalence ^12, 29^. The development and introduction of nucleic acid amplification test (NAAT) technologies in the 1990s provided commercially available laboratory systems, which are highly accurate, robust for the detection of *C. trachomatis*, *N. gonorrhoeae* and *T. vaginalis* in both urine and genital swabs, and have relatively short turnaround time. The costs and technical requirements of these platforms however have meant that they are not routinely available in resource-limited settings. In addition, despite the recognised consequences of gonorrhoea, chlamydia, trichomonas and bacterial vaginosis in pregnancy, there has been no consensus as to whether it is beneficial to offer antenatal screening and treatment for these infections. Authorities in Australia ^30^, and the United States ^31, 32^ recommend antenatal screening for chlamydia in all women under 25 years of age, but routine screening for asymptomatic gonorrhoea, trichomonas and bacterial vaginosis is not currently recommended, except for settings of increased risk, such as in remote Aboriginal communities in Australia.

In the absence of accurate diagnostic tests for STIs in high-burden, low-resource settings, presumptive strategies that do not depend on knowing a woman’s true infection status have been considered. A randomised trial of single dose presumptive STI treatment (1 g azithromycin, 400 mg cefixime and 2 g metronidazole) among pregnant women in Uganda found significant reductions in low birth weight and neonatal death ^33^, but a subsequent sub-analysis found excesses of the same outcomes in the treatment arm among the infants of women who were retrospectively determined to have had trichomonas ^34^. This finding was consistent with an earlier trial in which women were presumptively given 2 g metronidazole twice during their pregnancy ^35^ and which found an increase in low birth weight; but not with another that used the same metronidazole dosing regimen to treat women with microscopically-confirmed bacterial vaginosis and found no such effect on birth weight ^36^. Conversely, in a field trial of 300mg clindamycin twice daily for 7 days for microscopically-confirmed bacterial vaginosis, the risk of preterm birth among women with bacterial vaginosis in the intervention phase was half that of women with this condition in the pre-intervention phase (RR 0.5; 95% CI: 0.3-0.9) ^28^. In contrast to earlier research that suggested treatment for trichomonas in pregnancy might increase the risk of adverse birth outcomes (hypothesized to be the result of dying trichomonads releasing inflammatory mediators thereby inducing preterm labour) ^31, 37^, a trial among 2428 women in South Africa found no association between oral metronidazole treatment and preterm birth among women diagnosed with trichomoniasis ^38^; as did a recent retrospective cohort study among over 4000 women in the United States ^39^. Faced with this conflicting body of scientific evidence, recent reviews have concluded that there is insufficient evidence to inform policy on STI testing and treatment in pregnancy, and that field trials are needed to confirm or refute earlier findings on the potential risks and benefits ^31, 32, 40^.

New point-of-care diagnostic technologies for STIs have become available in the last 5 years and make it possible for the first time to conduct such trials in high-burden, low-resource settings. Following the successful implementation of point-of-care devices for the rapid diagnosis of syphilis and HIV, there was an effort to use similar strategies, generally based on lateral flow technology, for chlamydia, gonorrhoea and trichomonas testing, but the results were disappointing, largely due to poor sensitivity ^41– 46^. In the past 5 years, there has been a major breakthrough in rapid diagnosis of STIs. A fully automated portable NAAT platform (GeneXpert, Cepheid, Sunnyvale, CA) can perform tests for *C. trachomatis*, *N. gonorrhoeae* and *T. vaginalis* that are as accurate as laboratory-based NAATs ^47– 50^. This platform has substantially improved the diagnosis and management of tuberculosis in many low- and middle-income settings, including PNG ^51^. Disposable cartridges hold the reagents including primers and probes for the simultaneous detection of chlamydia and gonorrhoea, with a separate cartridge used for trichomonas detection. Test results are available in approximately 90 minutes for Xpert CT/NG and 60 minutes for the Xpert TV test. Building on the experience from the Test Treat ANd GO (TTANGO) Trial ^52^ in remote Aboriginal communities in Australia, we showed the feasibility of point-of-care STI testing and treatment in a pilot study in selected antenatal settings in PNG ^52, 53^. The diagnosis of bacterial vaginosis has until recently relied on Amsel’s clinical criteria, or on highly skilled, time-consuming microscopic examination of Gram stained vaginal smears using the Nugent score ^53– 55^. The BVBlue test (OSOM BVBlue Test, Gryphus Diagnostics, USA) is a chromogenic test based on the detection of increased vaginal fluid sialidase activity ^56^. It is the first robust point-of-care test available for the diagnosis of bacterial vaginosis and, in previous evaluations, had high sensitivity (90%) and specificity (95%) compared with clinical and laboratory criteria ^56, 57^.

### Significance and potential impact of the trial

Women in LMIC worldwide experience a high burden of adverse birth outcomes. The body of available scientific evidence about the effect of testing and treatment for STIs and other genital infections in pregnancy is conflicting. Recent reviews have concluded that there is insufficient evidence to inform policy on STI testing and treatment in pregnancy, and that field trials are needed to confirm or refute earlier findings on the potential risks and benefits ^31, 32, 40^. If we show a benefit for antenatal point-of-care STI testing and treatment, our findings could hasten access to these technologies and thereby lead to improved maternal and neonatal health. If no benefit is found, resources for maternal and neonatal health care can be directed elsewhere.

## Protocol

### Trial aim and objectives

The overall aim of the Women And Newborn Trial of Antenatal Interventions and Management (WANTAIM) is to measure the effectiveness, health system implementation requirements, cost-effectiveness and acceptability of antenatal point-of-care STI testing and immediate treatment to improve birth outcomes in high-burden, low-income settings. 

*Primary Objective*

1. Evaluate whether point-of-care testing and immediate treatment of curable STIs in pregnancy leads to a reduction in preterm birth and/or low birth weight compared with standard antenatal care.

In this trial, ‘curable STIs’ includes *C. trachomatis*, *N. gonorrhoeae*, *T. vaginalis* and bacterial vaginosis, all of which will be tested for and treated at point-of-care in the intervention arm.

*Secondary Objectives*

1. Evaluate whether point-of-care STI testing and treatment in pregnancy leads to an increase in mean birth weight compared with standard antenatal care;

2. Evaluate whether point-of-care STI testing and treatment in pregnancy leads to a reduction in premature rupture of membranes compared with standard antenatal care;

3. Evaluate whether point-of-care testing in pregnancy increases the diagnosis and treatment of STIs compared with symptom-based ‘syndromic’ management;

4. Evaluate the cost-effectiveness of point-of-care STI testing and treatment in pregnancy compared with standard antenatal care;

5. Evaluate the health system implementation requirements of point-of-care STI testing and treatment in pregnancy compared with standard antenatal care;

6. Evaluate the acceptability of antenatal point-of-care STI testing and treatment compared with standard care;

7. Evaluate whether point-of-care STI testing and treatment in pregnancy leads to a reduction in neonatal eye infection and/or pneumonia compared with standard antenatal care ( *among a sub-set of 2000 participants only*);

8. Evaluate mother-to-child transmission of *C. trachomatis* and *N. gonorrhoeae* ( *among a sub-set of 2000 participants only*);

9. Evaluate the performance of the Xpert ^TM^ CT/NG Test for the diagnosis of neonatal eye infection and pneumonia using ocular and nasopharyngeal specimens ( *among a sub-set of 2000 participants only*).

### Trial outcome measures

*Primary*

The primary outcome is a composite measure of two events, the proportion of women and their newborns in each trial arm who experience either:

a) preterm birth (live birth before 37 weeks’ gestational age as estimated by ultrasound examination at 26 weeks’ gestational age or earlier, adjusted according to reported date of last menstrual period in accordance with trial standard operating procedures); and/or

b) low birth weight (birth weight <2500 g measured as soon as possible after birth using calibrated, medical-grade infant weighing scales accurate to within 10 g; birth weights measured within 72 hours of birth only will be included in the primary outcome).

*Secondary*

The trial secondary outcome measures are:

1. Mean birth weight among newborns in the control and intervention arms of the trial;

2. Proportion of women who experience premature rupture of membranes (defined as membrane rupture before the onset of labour);

3. Number of curable STIs diagnosed and treated;

4. Incremental cost effectiveness ratios (cost per preterm birth and/or low birth weight case averted; cost per STI diagnosed and treated; cost per life year saved; cost per disability-adjusted life year (DALY) averted);

5. Health system implementation requirements;

6. Acceptability of antenatal point-of-care STI testing and treatment among (a) women and (b) health workers.

7. Proportion of newborns with an eye infection or moderate/severe pneumonia by 4–6 weeks postnatal ( *among a sub-set of 2000 participants only*);

8. Incidence of mother to child transmission of *C. trachomatis* or *N. gonorrhoeae* as indicated by positive newborn eye ( *C. trachomatis* or *N. gonorrhoeae*) or nasopharyngeal ( *C. trachomatis*) swabs by 4–6 weeks postnatally ( *among a sub-set of 2000 participants only*);

9. Diagnostic test accuracy (sensitivity, specificity, positive and negative predictive values) of Xpert CT/NG Test compared with laboratory-based polymerase chain reaction (PCR) assays ( *among a sub-set of 2000 participants only*).

The health economics and health systems implementation components of the trial will be described in detail in separate protocol papers.

### Trial design and setting

*Rationale*

WANTAIM is a cluster-randomised controlled crossover trial ^58^ being conducted in 10 clusters in PNG. We have adopted a cluster randomised trial design as our preferred option. Individual randomisation cannot be considered because it is neither logistically nor ethically feasible to randomly assign women to different types of care within the same clinic. In any case, the intervention cannot be blinded or compared with a placebo, so there is substantial potential for bias due to differential provision of care in the two arms, even if individual randomisation could be used.

A crossover design was chosen rather than a parallel cluster randomised trial design due to the considerable statistical efficiency of the former, and because this design provides an opportunity for all clusters to participate in the intervention arm at some point during the trial. In addition, this design will allow the health systems, health economics and acceptability components of the trial to be conducted in the same settings under different trial conditions (enabling direct ‘before / after’ comparisons to be made).

*Selection of trial clusters*

Trial clusters have been selected in consultation with provincial health authorities, church health services, health facility staff and other local stakeholders. Sites were selected based on antenatal clinic attendance data (e.g. average number of new attendees per week; total new attendees in previous 12 months; proportion of new attendees who met trial eligibility criteria); clinic staffing, experience and interest in participating in the trial; available clinical space and infrastructure (e.g. to enable point-of-care STI testing and treatment, ultrasound scans, and other trial procedures to be carried out); geographical location of health facility and catchment communities (in relation to other potential trial clusters); and previous experience in PNGIMR-led clinical and community-based research.

Each of the selected cluster health facilities has a designated weekday for new antenatal attendances, during which an average of 12–18 women attend their first antenatal visit. Site assessment visits indicated that around 70% of those attending would be eligible to participate in the WANTAIM trial. Assuming a high proportion (85%) of women who are eligible elect to participate, each site will enrol around 7–10 women per week and take 23–33 weeks to recruit the required sample size of 230 women per cluster in each phase of the trial.

*Intervention summary*

Women participating in the intervention arm of the trial will provide self-collected vaginal specimens for point-of-care STI testing (chlamydia, gonorrhoea, trichomonas and bacterial vaginosis), and will be provided with immediate treatment as indicated by their test results, at the following time points:

•   At enrolment (preferably before 20 weeks gestation);

•   One month after trial enrolment (to confirm that infections at enrolment have been cured and to detect incident infections. Women with a positive test result at this visit will be asked to return for repeat testing one month later);

•   At 34–36 weeks antenatal follow-up.

The rationale for this intervention schedule is based on:

a)   current scientific evidence which suggests that diagnosis and treatment of chlamydia, gonorrhoea, trichomonas and bacterial vaginosis early in pregnancy would have the greatest impact on low birth weight and preterm birth ^8, 31^;

b)   the lack of scientific evidence on the association between incident chlamydia, gonorrhoea, trichomonas and bacterial vaginosis in later pregnancy and risk of adverse birth outcomes, particularly preterm birth and premature rupture of membranes ^31, 40^.

To evaluate whether point-of-care testing in pregnancy increases the diagnosis and treatment of STIs compared with symptom-based syndromic management, residual urinalysis specimens collected at enrolment, after one month and at 34–36 weeks in the control arm of the trial will be retained and tested in batches. This will also enable the research team to provide appropriate antibiotic treatment at the postnatal visit, as indicated.

**Figure 1.**
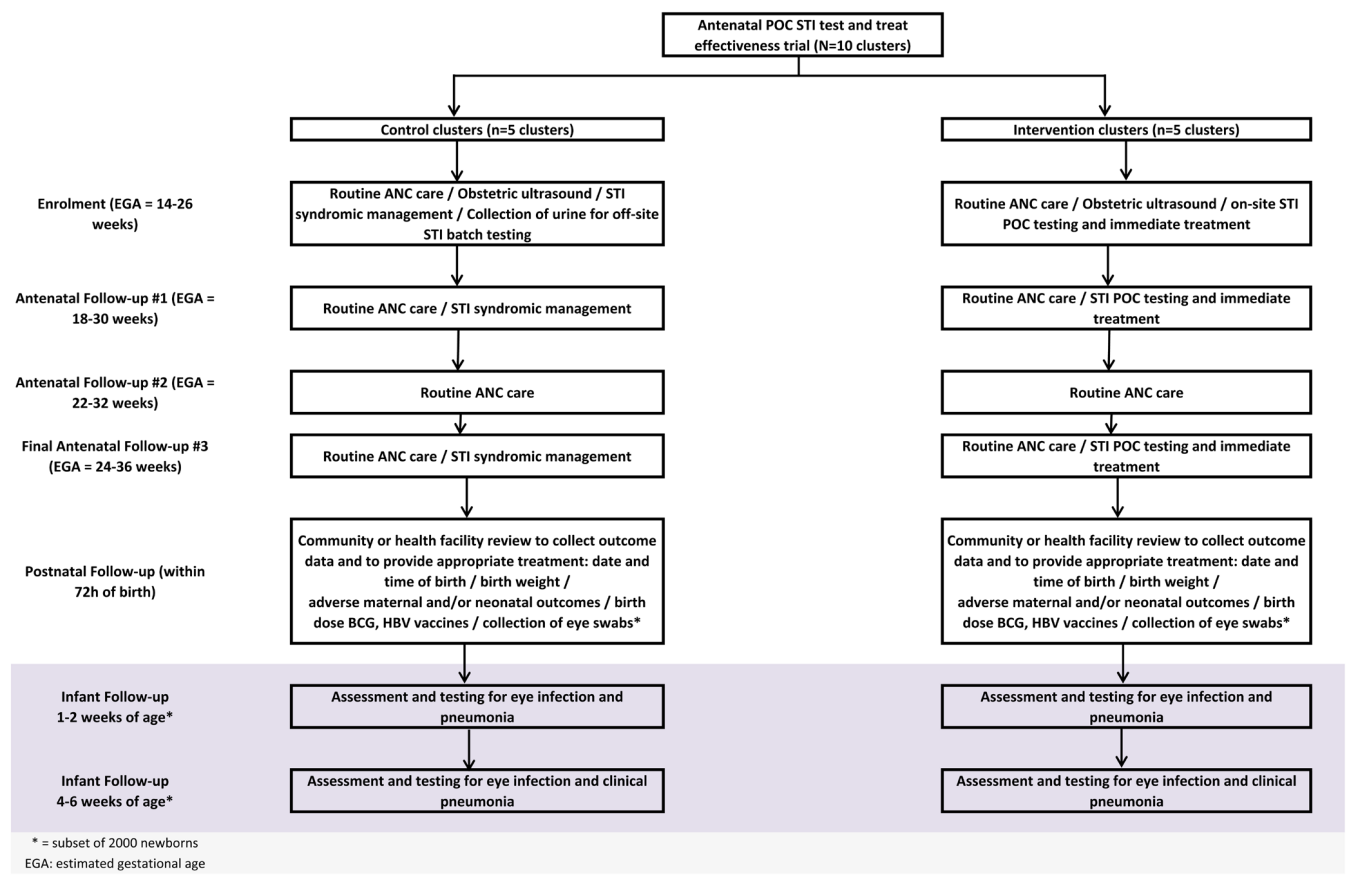
Summary of trial interventions and visits.

**Figure 2.**
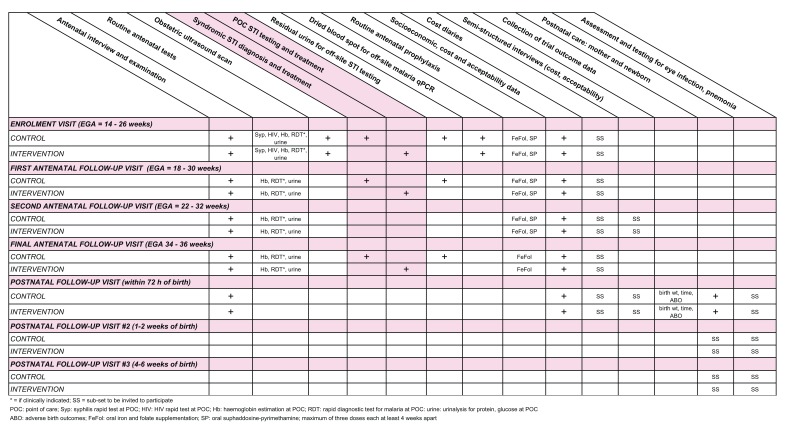
Summary of trial procedures.

A summary of trial interventions and visits in Figure 1 and a summary of trial procedures is provided in Figure 2.

### Randomisation

The unit of randomisation is a primary health care facility and its catchment communities. Ten geographically distinct clusters will be assigned in a 1:1 ratio to control and intervention arms in the first phase of the trial. Following a washout period of 2–3 months at the end of the first phase, each cluster will crossover to participate in the alternative trial arm in the second phase of the study (a so-called ‘A-B / B-A’ trial design). Randomisation will be stratified by province. In each province close to the start of recruitment, members of the local community advisory board, representatives from local health authorities, and staff from clinical research sites will be invited to a launch event in which the randomisation sequence will be selected by a senior independent stakeholder e.g. the Provincial Administrator. The Chief Investigator will prepare different randomisation sequences that will be put into separate identical opaque envelopes. These will be placed in a traditional woven string bag (a *bilum*). The independent stakeholder will select one envelope from the *bilum* and open it to reveal the final sequence to the audience.

A subgroup of up to 2000 newborns will be followed up at 1–2 weeks and 4–6 weeks postnatal to collect secondary outcome data (secondary objectives 7–9). Five of the 10 trial clusters will be randomly selected to participate in this part of the study. The randomisation sequence will be computer-generated and allocated by the trial statistician. Each of the clusters selected to participate in the extended postnatal follow-up component will do so during both the intervention and the control phase of the trial. Each selected cluster will thereby contribute around 200 newborns in each trial phase (2000 in total).

### Study participants and eligibility criteria

Women aged 16 years or over, attending antenatal clinic at 26 weeks gestation or below (confirmed by obstetric ultrasound scan at first antenatal clinic visit), living within approximately one hour’s drive of a participating study clinic, and able to provide reliable contact details to facilitate future community tracing and postnatal follow-up, are eligible to join the study. Women found to be severely anaemic at enrolment (haemoglobin less than 6.0 g/dl accompanied by symptoms), or those with a permanent disability that prevents or impedes study participation and/or comprehension (such that it is not possible to obtain informed consent to participate), are excluded from participation in the trial. All those excluded on health grounds will be referred to the appropriate clinical specialist at each site, where they will be managed according to PNG national treatment guidelines ^59^.

### Field team

Overall responsibility for the conduct and management of the trial will rest with the Chief Investigators. The study will be managed on a day-to-day basis by an experienced Trial Coordinator based at the PNGIMR who will oversee the trial at all sites and supervise a dedicated field team comprising clinical, laboratory, data management, community liaison and administrative support staff. Clinical research staff will be qualified nurses and/or midwives, and will be responsible for the conduct of trial-related procedures and the provision of routine antenatal services for women participating in the trial.

### Recruitment and informed consent

Immediately prior to the start of enrolment at each site, potential participants will be notified that the trial is about to start through community meetings (e.g. at markets, after church), local media (e.g. broadcasts in PNG Pidgin [one of three official national languages in PNG], and local languages), and other mobilisation activities as advised by local stakeholders. Activities will be conducted by clinical research staff and members of the study community liaison team. On arrival at participating antenatal clinics, women will be provided with general information about the trial though a 5–10-minute group talk provided by a member of the clinical research team using a pictorial flipchart. Women who express an interest in taking part in the trial will be given a copy of the study Participant Information Sheet (available on Open Science Framework ^60^) to read whilst waiting.

All participants will be required to complete written informed consent procedures prior to trial enrolment. All information and consent procedures will be conducted in PNG Pidgin or English, as preferred by the individual participant. In accordance with ICH Good Clinical Practice guidelines (ICH GCP), participants unable to read and/or write will be required to have an impartial witness present during the above procedures.

### Enrolment visit procedures

Women attending routine antenatal clinics in both the control and intervention arms of the trial will receive standard antenatal care in accordance with PNG national guidelines ^59^. All women will be advised to return for a minimum of three additional antenatal clinic visits, in accordance with national and WHO recommendations; and to attend a health facility to give birth.

*Procedures at enrolment: control clusters*

a) The following routine antenatal clinic procedures will be conducted ^59^:

○ A face-to-face clinical interview conducted in the most appropriate language (e.g. English or PNG Pidgin) to collect baseline antenatal data, including current and past obstetric history.

○ A general medical and obstetric examination, in which the following will be recorded: general health and well-being; signs of anaemia or other systemic disease (e.g. tuberculosis); weight and height; blood pressure; fundal height and foetal heart rate.

○ Provider initiated counselling and testing for HIV (PICT) and syphilis infection, as per PNG national guidelines.

○ Collection of specimens for routine on-site testing: haemoglobin (Hb) estimation; rapid diagnostic test (RDT) for malaria (if symptomatic e.g. fever); collection of a midstream urine specimen for urinalysis (protein and glucose testing).

○ Women will be provided with routine antenatal care as per PNG national guidelines, including sulphadoxine-pyrimethamine for malaria prevention; iron and folate supplementation; tetanus toxoid immunization; treatment for syphilis if required; and STI syndromic management for women presenting with genital symptoms, as per PNG national guidelines.

○ Women with a positive RDT for malaria will be given malaria treatment in accordance with PNG national treatment guidelines.

○ Women who test positive for HIV infection will be referred for specialist review and initiation of antiretroviral therapy.

b) The following study-specific procedures will be conducted:

○ Collection of additional clinical, behavioural and socio-economic and demographic information, including risk factors for low birth weight and preterm birth (e.g. smoking, alcohol and betel nut consumption; recent or current systemic illness, e.g. malaria or tuberculosis; poor maternal nutritional status, e.g. mid-upper arm circumference <22 cm) ^61– 63^

○ A trans-abdominal obstetric ultrasound examination to measure biparietal diameter (BPD), head circumference (HC), femoral length (FL) and abdominal circumference (AC), to estimate gestational age, as specified in study-specific standard operating procedures.

○ Storage of residual urine specimen for off-site STI PCR testing to be conducted in batches in order to obtain information on the prevalence of curable STIs ( *C. trachomatis, N. gonorrhoeae, T. vaginalis* and bacterial vaginosis) among women participating in the control arm. Urine remaining after off-site STI testing will be retained for future laboratory testing with participants’ written consent.

○ Women with a positive urine test result will be counselled regarding their diagnosis and provided with treatment for them and their partner at the first postnatal visit conducted within 72 hours of birth.

○ A dried blood spot (DBS) will be collected and stored for later off-site malaria quantitative real-time PCR (qPCR) testing.

○ Information will be recorded in a study-specific Enrolment Case Report Form (CRF) at the time the measurements and interview are conducted.

○ A summary of key clinical findings will also be recorded in the participant’s Health Record Book that will be checked to see if any visits to a health centre have been documented. If the participant has any record of such a visit, the field study team will record the date of the most recent visit and any medication the participant received in the Enrolment CRF.

○ At the end of the Enrolment visit, a short interview with a dedicated community volunteer will be conducted in order to collect home address and other locator information to facilitate future community tracing and postnatal follow-up. A separate Locator Form will be used to record this information and will be securely stored in a different location to completed Enrolment CRFs.

*Procedures at enrolment: intervention clusters*

Enrolment procedures in intervention clusters will be identical to those in control clusters, with the following exceptions:

○ Women will provide two self-collected vaginal swabs for same-day, point-of-care testing at the study health centre:

a) one swab will be tested for chlamydia, gonorrhoea and trichomonas using the GeneXpert platform; and
b) one swab will be tesed for bacterial vaginosis, using the BVBlue Test.

○ Clinic staff will use a laminated pictorial guide to help them explain the correct way to collect vaginal specimens for STI testing. During the explanation, staff will indicate how samples are to be collected using a specimen collection kit reserved for this purpose, and will encourage women to handle the swabs and to review the pictorial guide themselves.

○ When staff are satisfied that the participant understands the collection procedure, she will be asked to collect her specimens in a private room or the clinic toilet. This approach has been successfully used in our earlier research and found to be acceptable to both study participants and clinic staff.

○ Women who have a positive STI test result will be provided with immediate, directly observed treatment in accordance with PNG national guidelines for the management of STIs. Women will also be counselled regarding the importance of providing STI treatment for their husband/partner in order to prevent re-infection, and the most appropriate options for ensuring successful partner treatment (e.g. same-day treatment if accompanied by their partner; provision of a partner treatment pack; return for couple counselling and treatment).

○ Only those with a positive STI test result will be treated. STI syndromic management will only be provided for vulvovaginitis considered on clinical features due to candidiasis, for which additional treatment will be provided as per national guidelines (e.g. nystatin pessaries and/or clotrimazole cream) ^59^.

○ If the test result on any of the above assays is invalid, women will be asked to provide an additional self-collected swab and given further counselling on self-collection, to allow repeat testing on the same day in the clinic.

○ Residual fluid remaining in the GeneXpert sample processing tube will be retained for future laboratory testing with participants’ written consent.

○ Urinalysis will be conducted as per routine antenatal care (e.g. for protein and glucose) but residual urine will not be stored in intervention clusters.

### Procedures during antenatal follow-up

Following enrolment, women in both the control and intervention arms of the trial will receive standard routine antenatal care and will be asked to re-attend for a minimum of three further antenatal clinic visits, in accordance with national guidelines:

1) The first antenatal follow-up visit will be conducted approximately four weeks after trial enrolment;

2) The second antenatal follow-up visit will be conducted approximately eight weeks after trial enrolment;

3) The third antenatal follow-up visit will be conducted in the third trimester at approximately 34–36 weeks gestation.

*Procedures during antenatal follow-up: control clusters*

○ A general medical and obstetric examination, and conduct of routine antenatal investigations: haemoglobin estimation; RDT for malaria (if clinically indicated); collection of a midstream urine specimen for urinalysis (protein and glucose testing).

○ In addition to routine antenatal care, a short face-to-face interview will be conducted at all visits to obtain information on risk factors for low birth weight/preterm birth (e.g. smoking, alcohol and betel nut use) and potential confounders (e.g. care-seeking, sexual behavioural data, condom use, concomitant antibiotic use for other non-genital infections).

○ Information will be recorded in study-specific CRFs at the time the measurements and interview are conducted.

○ Participants will be provided with health information at all visits, particularly the importance of having a supervised birth in a health facility, and reminded of the importance of informing the research team within 24 hours of giving birth if they have been unable to give birth at their local health facility.

○ All those who present with symptoms suggestive of a genital STI will be treated according to national syndromic management guidelines.

○ At follow-up visits #1 and #3, a urine specimen will be collected and stored for off-site STI testing to be conducted in batches in order to obtain information on the prevalence of curable genital STIs among women participating in the control arm. Women who test positive for these infections (and their partners) will be provided appropriate treatment at their first postnatal visit.

*Procedures during antenatal follow-up: intervention clusters*

○ Follow-up procedures in intervention clusters will be identical to those in control clusters with the exception that at follow-up visits #1 and #3, women will be asked to provide two self-collected vaginal swabs that will be tested as per the enrolment visit. Women who have a positive STI test result provided with same-day treatment in the clinic.

### Postnatal follow-up procedures

Women participating in the control arm of the trial who have a positive urine STI test result during antenatal follow-up will be provided with appropriate treatment by the study team at the first postnatal visit. Procedures for postnatal follow-up visits will otherwise be the same in each trial arm. Eye and nasopharyngeal swabs will be collected from a sub-set of up to 2000 newborns in five randomly selected trial clusters to address secondary objectives 7–9.

*First postnatal follow-up visit (within 72 hours; all participants)*

○ A first postnatal follow-up visit will be conducted by the trial team as soon as possible after birth to collect primary outcome data (birth weight, date and time of birth); and clinical information, including adverse maternal and newborn health outcomes. Data will be recorded in study-specific CRFs at the time the measurements and interview are conducted. A summary of key findings will also be recorded in the participant’s client-held Health Record Book.

○ Participants who give birth at a health facility will be visited at the facility by a research nurse/midwife. Those who give birth in the community will be visited at home by a study community nurse/midwife.

○ The trial team will aim to conduct all first postnatal visits within 72 hours of birth. Baby weight measured in postnatal visits conducted more the 72 hours but less than 1 week after birth will be categorised as ‘late birth weight’ and will not contribute to the primary outcome.

○ Birth weight data recorded by labour ward staff in client-held Health Record Books at the time of childbirth will be captured at postnatal visits conducted by the study team. If the study team have been unable to conduct a postnatal visit within 72 hours of birth, the birth weight as recorded in the client-held record book will be used in statistical analyses and contribute to the primary outcome of the trial. All health facilities participating in the trial (and other local facilities where study participants may give birth, such as provincial hospitals) will be provided with medical grade infant weighing scales accurate to within 10 g. Scales used by the study team and those at local health facilities will be calibrated monthly in accordance with study-specific standard operating procedures (SOPs).

○ Adverse maternal and/or neonatal health outcomes will be treated in the community and/or referred to local health services as indicated, in accordance with PNG national guidelines.

In clusters randomised to take part in the extended neonatal component of the trial, two eye swabs (one from each eye) will be collected from babies at this visit (for comparison of GeneXpert CT/NG Test and a laboratory-based NAAT platform)

*Second postnatal visit (1–2 weeks; sub-set of 2000 participants only)*

○ A second postnatal visit will be carried out in the participant’s home to collect information on adverse maternal and newborn health outcomes (including neonatal eye and respiratory symptoms) and health service utilisation.

○ A neonatal examination will be conducted and baby weight measured as above. Adverse maternal and/or neonatal health outcomes will be treated in the community and/or referred to local health services as indicated, in accordance with PNG national guidelines.

○ The following specimens will be collected at this visit:

• Two eye swabs from neonates with any clinical signs of conjunctivitis and from a random 15% sample of neonates with no clinical signs of conjunctivitis (one for GeneXpert CT/NG Test, and one for storage and later testing using a laboratory-based PCR platform)
• Two nasopharyngeal swabs (one for Xpert CT/NG Test, and one for storage and later testing using a laboratory-based PCR platform)

○ Neonates with a positive test result will be followed-up in the community and provided with appropriate antibiotic treatment in accordance with PNG national guidelines.

○ Data collected during the visit (and any treatment provided and/or referral initiated) will be recorded in study-specific CRFs at the time of the visit. A summary of key findings will also be recorded in the participant’s Health Record Book.

*Third postnatal visit (4–6 weeks; sub-set of 2000 participants only)*

○ A third postnatal visit will be conducted home to collect information on adverse maternal and newborn health outcomes (including neonatal eye and respiratory symptoms) and health service utilisation. Visits will be conducted at participating health facilities when mothers and their babies attend for routine infant diphtheria-tetanus-polio (DTP) vaccination, or at home if participants miss a routine vaccination clinic.

○ Data will be extracted from client-held health record books and health facility records of investigations, diagnoses and treatment where available.

○ Procedures for the collection of health information, and treatment provided will be the same as at the second postnatal visit, described above.

○ The following specimens will be collected at this visit:

• Two nasopharyngeal swabs (one for Xpert CT/NG Test, and one for storage and later testing using a laboratory-based PCR platform).
• Eye swabs will not be collected unless clinically indicated at this visit.

○ Infants with a positive test result will be followed-up in the community and provided with appropriate antibiotic treatment in accordance with PNG national guidelines.

### Collection of economic evaluation data

The cost effectiveness analysis will be performed according to the intention-to-treat principle and from a health service and societal perspective. It will measure and value the resource use of providers and participants. This includes all costs of the intervention, including local health service implementation net costs (such as the costs of service provision, medicines, diagnostic tests, changes to staff time, other aspects of workflow, and supervision, adjusting for any user fees observed); participant out-of-pocket costs (such as transport, accommodation); and costs of production losses (such as participant’s and family’s time-off work or normal activities). The project costs of delivering the intervention will be collected retrospectively from the project accounts and entered into a standardised data capture tool designed for this purpose following standard practice ^64^. Costs incurred by health care providers (i.e. participating health facilities) will be collected prospectively from health service records (below) that capture data on changes in service provision, patient flow, staff allocation of time, and resource use (drugs and equipment); combined with available financial data on the average unit cost of delivering the services in both arms of the trial ^65^. Participant costs will be assessed prospectively using a context-specific client CRF covering cost of care-seeking that will be administered by research staff to all participants at enrolment, at antenatal follow-up visits and postnatally. This CRF will also include questions to assess client acceptability of the intervention (see below). The health economics research component of the trial will be described in detail in a separate protocol paper.

### Collection of health system implementation data

All trial facilities will be assessed at baseline and up to twice during implementation to collect health service data through examination of facility records, observation of facility procedures, and semi-structured interviews (SSIs). SSIs will collect provider costs (capital and recurrent) and other health service feasibility and acceptability data, including changes in service provision, patient flow, staff allocation of time, and resource use (drugs and equipment). SSIs will be conducted with members of the in-country clinical research team (1–3 staff per cluster; 10–30 interviews in total); clinic staff in participating antenatal clinics (1–3 staff per facility; 10–30 interviews in total); and health managers at district, provincial, national level (10–15 interviews in total). Health care managers and providers being interviewed or observed will complete separate informed consent procedures, and interviews digitally recorded with participants’ consent. Interview topics will include:

○ Rapid health system screen for changes in antenatal service delivery, facility staffing, health information, financing and payments, clinical governance, and community/patient engagement.

○ Staff workflow and patient flow specific to the conduct of point-of-care STI testing, counselling and treatment.

○ Provider views on training requirements, confidence in test results obtained, experiences of providing results to participants.

○ Provider views on resources needed in terms of capital and recurrent costs to health service and clients, staffing and infrastructure.

○ Provider views on changes in client attitudes to and utilization of antenatal services, including frequency and timeliness.

○ Opinions on enablers of and barriers to deployment of antenatal point-of-care STI testing in general and GeneXpert-based point-of-care testing in particular.

An observational assessment and an examination of facility records will also be conducted at the time facilities are visited for SSIs. The observational assessment comprises a structured observation of clinic processes, and will include quantification of staff work-flow and patient-flow. Observational assessments will be conducted at baseline, and at early and late time points during trial implementation at clinical sites. The examination of facility records will review clinic activity levels relevant to the study including staff time and other resource allocations, utilisation patterns, timeliness of antenatal care, clinical complications of pregnancy and timing of diagnosis and treatment. The health systems research component of the trial will be described in detail in a separate protocol paper.

### Collection of acceptability data

Client acceptability of the intervention, and accompanying service changes, will be measured in two ways. First, questions embedded in the CRF on cost of care-seeking (see above) will be administered by research staff to all participants at enrolment, and antenatal and postnatal follow-up visits. These data will be supplemented by SSIs (coordinated with the SSIs conducted above) in a subset of up to 200 women, enabling qualitative triangulation of issues raised in other data collection activities. Interviews will be administered by research staff trained in culturally sensitive collection of qualitative information. Due to the iterative, validating role of these SSIs, their structure and themes will be revised (within the confines of the outline interview guide approved by research ethics committees) based on early findings from acceptability CRFs and provider interviews.

### Adverse events and safety reporting

***Reporting framework.*** Adverse Events (AEs) occurring during the course of the trial will be monitored, managed and reported in accordance with guidance from the UK Medical Research Council, and the UK National Health Service Health Research Authority, in particular their guidance on safety reporting in human research trials that are not clinical trials involving investigational medicinal products (non-CTIMP trials: https://www.hra.nhs.uk/approvals-amendments/managing-your-approval/safety-reporting/). The WANTAIM trial is evaluating the use of established STI diagnostic assays and providing the same antibiotics for the treatment of infections as currently used for STI syndromic management in PNG. No new investigational medicinal products are being evaluated in the trial.

***Protocol definition of Serious Adverse Event.*** The protocol definition of a serious adverse event (SAE) is based on UK guidance for non-CTIMPs trials ( Box 1). This definition is consistent with ICH GCP and current MRC UK guidelines. All SAEs as defined will be reported within 72 hours of the Trial Coordinator and/or Chief Investigator becoming aware of the event.

Box 1. **Protocol definition of serious adverse event.**

**In research other than CTIMPs, a serious adverse event (SAE) is defined as an untoward occurrence that:**

(a) results in death;

(b) is life-threatening;

(c) requires hospitalisation or prolongation of existing hospitalisation;

(d) results in persistent or significant disability or incapacity;

(e) consists of a congenital anomaly or birth defect; or

(f) is otherwise considered medically significant by the investigator.**

**An SAE occurring to a research participant should be reported to the main Research Ethics Committee where in the opinion of the Chief Investigator (CI) the event was:**

Related – that is, it resulted from administration of any of the research procedures, and

Unexpected – that is, the type of event is not listed in the protocol as an expected occurrence.

** In the WANTAIM trial, the following conditions will be considered medically significant and categorized as SAEs:

spontaneous abortion / miscarriage

stillbirth

early neonatal death

domestic violence resulting in injury that requires medical attention

### Sample size

Sample size requirements were based on our earlier findings about the incidence of the primary outcome in PNG ^13– 15, 66^. The proportions of pregnancy resulting in preterm birth and low birth weight were estimated to be 15% each, and 18% combined. Research findings on the relationship between STIs and birth outcomes suggest that effective STI treatment could reduce this combined proportion by up to 45% ^27, 33^ in those with STIs. This reduction would translate into a relative reduction of around 23% in a population in which nearly half of all pregnant women have chlamydia, gonorrhoea, trichomonas and/or bacterial vaginosis, as is the case in PNG ^14^. To measure this effect size with α=0.05, β=0.20 (80% power), and an estimated intra-cluster correlation coefficient (ICC) of 0.003, 16 clusters of 200 women would be required in a cluster randomised crossover trial (8 clusters per phase; 3200 women in total). In case one or more trial cluster does not successfully complete the trial, two additional clusters will be added (10 clusters of 200 women in each phase; 4000 women in total). Based on our earlier work in this setting ^66– 68^, we anticipate loss to follow-up for the primary outcome of around 10–15%, so we will recruit a total of 4600 women from 10 clusters in two phases (230 women per cluster in each phase of the trial).

### Methods for minimising bias

To minimise the risk of selection bias due to differences in the proportion of eligible women enrolled in larger compared to smaller clinical centres:

○ Human resource allocation, equipment, consumables and logistics support will be increased in clusters having the highest weekly attendance figures to facilitate optimal participant accrual across all sites.
○ Site-level accrual will be monitored weekly by the Trial Coordinator and presented at quarterly Trial Management Group (TMG) meetings held throughout the course of the trial. The total number of women attending their first antenatal clinic visit, total enrolled, total excluded, and total who declined to join the study, will be recorded in a weekly tally sheet at each site.

Attrition bias in the ascertainment of outcomes will be minimised by the use of previously tested strategies for ensuring high levels of follow up. To minimise performance bias we will be using the same procedures for routine antenatal care in all clinics. Detection bias will be minimised by assessing outcomes as objectively as possible and by blinding assessments wherever possible.

### Statistical analysis

The statistical analysis plan for the trial will be presented in detail in a separate manuscript.

All analyses (including the cost-effectiveness analysis) will be conducted using methods that take into account the cluster-randomised cross-over design. All analyses will be carried out using the latest available version of Stata statistical software (15.0 or above; College Station, TX, USA).

Primary data analysis will be by intention to treat (ITT) at the cluster level, meaning that all participants with a recorded outcome will be included in the analysis, and will be analysed according to the treatment group to which they were randomised; and all clusters will be included in the analysis according to the treatment group to which they were randomised. Initial analyses will be unadjusted comparisons of intervention and control clusters. If there appear to be any important imbalances between randomised groups in terms of baseline demographic characteristics, risk factors for low birth weight and/or preterm birth, and the prevalence of specific STIs, adjusted analyses will be performed, and presented in addition to unadjusted comparisons.

Effect measures, comparing outcomes between intervention and control clusters, will be estimated with 95% confidence intervals and p-values from the corresponding hypothesis tests. The effect measure for the primary outcome will be a risk ratio, defined as the ratio between the proportion recorded as having the outcome of interest in the intervention arm, and the corresponding proportion in the control arm.

### Data management

Data will be collected using paper-based CRFs at each visit (enrolment, antenatal follow-up and postnatal visits). All CRFs will be designed in TeleForm Elite version 10.5 and uploaded into a secure centrally located clinical trials database created in ORACLE. Completed CRFs will be checked on the day of completion and any errors, discrepancies or out of range values will be corrected by study staff. Any corrections or alterations to the original CRF will be initialled by the member of staff. Original copies of the CRF will be held at the clinic in a locked filing cabinet accessible only by the clinic staff and the dedicated trial Data Manager. For the purposes of ICH GCP, completed CRFs will be considered source documents. Completed CRFs will be electronically scanned, either at the clinic or at a central facility, on a weekly basis (e.g. the day following new antenatal enrolments) using a portable scanner into a computer using a tagged image file format (TIFF). Data will be stored in a dedicated electronic folder prior to verification using TeleForm. A copy of the locally-held data will then be uploaded using TeleForm into a dedicated study-specific ORACLE database located on a secure, off-site server at UNSW Sydney, Australia. ORACLE is compliant with ICH GCP E6 and with United States Food and Drugs Authority (FDA) guidance ^69^ (Title 21 of the Code of Federal Regulations (CFR) Part 11; Available at: http://www.fda.gov/RegulatoryInformation/Guidances/ucm125067.htm). UNSW Sydney will provide IT and technical support to the study team for the duration of the trial.

Following successful data upload, locally held data (TIFF scans) will be moved to a dedicated folder indicating completed data entry. Data entry and review will be ongoing activities during the entire trial period and will be conducted in accordance with study-specific SOPs. A random sub-set of electronic records from the master data base will be checked for accuracy against the scanned electronic TIFF files of participant CRFs every three months by the in-country trial Data Manager and Trial Coordinator. Electronic versions of the locked datasets and TIFF files for this study will be maintained as part of the PNG Institute of Medical Research (PNGIMR) research studies database. Electronic STI test results will be uploaded monthly into the ORACLE database as per study-specific SOPs, using a program developed at UNSW that enables test data to be extracted by Study ID Number from the .GXX data files generated by the GeneXpert platform. Electronic data held on portable ultrasound machines will also be regularly extracted for off-site back-up, as per study-specific SOPs.

The electronic trial database will be backed up both onsite (at PNGIMR) and off-site (at UNSW Sydney) at regular intervals as specified in study-specific data management SOPs. Copies of the databases generated in PNG and interim datasets will also be sent to the study CIs at regular intervals. At the end of the trial, the database will be locked following final data entry and database cleaning, and will then be provided to the Trial Statistician for data analyses. After completion of data analyses, copies of the final database and analytical datasets (neither of which will contain any subject identifying information) will be maintained on secure servers located at the PNGIMR and UNSW Sydney.

## Ethical considerations

### Confidentiality

Participant confidentiality will be maintained throughout the trial. All participants will be allocated a unique alphanumeric trial identification (ID) number that will be used to identify clinical and laboratory information in paper-based and electronic records to ensure confidentiality. Participants will not be identified by name. Names and identifier information will be recorded in the trial enrolment log (used to allocate trial ID numbers) and in study informed consent forms. These will be securely stored in a separate location to that used to store completed CRFs and laboratory records. Data uploaded to the centralised ORACLE clinical trials database will be identified only by trial ID number and participant initials.

### Informed consent

Women will be provided with general information about the trial on arrival at participating antenatal clinics though a 5–10 minute group talk provided by a member of the clinical research team. A pictorial informed consent flipchart will be used to supplement the talk and to explain key topics in more detail. The research team will not disclose information on whether the clinic is currently participating in the intervention or control arm of the trial. At the end of the talk, copies of the study participant information sheet will be provided to participants. Women who are interested to join the study will be assessed for eligibility by a member of the research team prior to formal informed consent procedures. Women who choose not to participate in the study or are ineligible will be assured of receiving standard care in full accordance with PNG standard treatment guidelines. Women will then be asked to complete formal written informed consent procedures that will include a short comprehension checklist completed by study staff to confirm that each participant understands key aspects of the trial prior to enrolment. In accordance with ICH GCP, participants who are unable to read and/or write will be required to have an impartial witness present during consent procedures. Example flipcharts in English and Pidgin, alongside informed consent forms, are available on Open Science Framework ^60^.

### Ethical approval

The trial protocol has been approved by the Institutional Review Board of the PNG Institute of Medical Research (IRB number 1608); the Medical Research Advisory Committee of the PNG National Department of Health (MRAC number 16.24); the Human Research Ethics Committee of the University of New South Wales (HREC number 16708); and the Research Ethics Committee of the London School of Hygiene and Tropical Medicine (REC number 12009).

### SPIRIT checklist

A SPIRIT checklist for the trial protocol is available from Open Science Framework ^60^.

## Trial governance and oversight

### Trial Steering Committee (TSC)

Primary responsibility for the conduct of the trial will rest with this committee that will be constituted according to MRC and ICH GCP guidelines ^70– 72^. Approved terms of reference for the TSC will be made available via the trial website. The TSC will meet prior to the start of the trial and then approximately twice a year to review progress, eligibility issues, adverse birth outcomes, and to consider any recommendations from the Data and Safety Monitoring Board (DSMB). The trial may be terminated by the TSC for any reason, including a recommendation of the DSMB. The TSC will be responsible for approving the trial protocol prior to its submission for national and international ethics committee review. Any protocol amendments or sub-studies to the main trial will also be submitted to the TSC for consideration prior to ethics approval. The TSC will also approve the trial monitoring plan and the statistical analysis plan prior to the start of participant enrolment; and be responsible for approving abstracts, conference presentations and manuscripts prior to their submission.

### Data and Safety Monitoring Board (DSMB)

The DSMB will oversee the trial and be comprised of senior researchers in maternal and child health, trial statisticians and provincial and national policy makers working in maternal health in PNG. The DSMB will provide expert advice and guidance to the TSC and will review primary trial outcome data every 4–6 months, including the possibility that point-of-care STI testing and treatment may be enhancing rather than reducing adverse birth outcomes. The DSMB will also review all SAE Reports. The DSMB will review incidence rates in the different study arms and advise the TSC on any revision of the sample size required during the course of the trial. Meetings will be coordinated by the Chief Investigator, and the Trial Statistician will supervise and prepare the tables in the format approved by the DSMB.

### Trial Management Group (TMG)

The day-to-day management of the trial will be overseen by the TMG, which will meet on a regular basis (every 2–4 weeks at the start of the trial and then quarterly) to review progress, including accrual and retention; eligibility and exclusions; STI diagnoses and treatment; data management issues; and logistical considerations. The TMG will also ensure that the routine operations of the trial proceed in accordance with the trial protocol and study-specific SOPs, and will engage with stakeholders and participating communities on an ongoing basis to provide progress updates on the conduct of the trial. TMG meetings will be coordinated by the Trial Coordinator and chaired by the Chief Investigator (CI).

### Trial monitoring

The trial will be independently monitored in accordance with MRC guidance and ICH GCP. As per ICH GCP E6, section 5.18.1, the purpose of trial monitoring will be to verify that: the rights and well-being of human subjects are protected; reported trial data are accurate, complete, and verifiable from source documents; and the conduct of the trial is in compliance with the approved protocol/amendment(s), with ICH GCP, and with applicable regulatory requirements. The Trial Monitor will have experience in monitoring large-scale clinical trials in similar settings to the current study and will be familiar with the trial protocol, relevant ethical and regulatory requirements, and study-specific SOPs. Prior to the start of the study, trial investigators will prepare a Monitoring Plan for endorsement by the TSC and the Trial Sponsor that will provide a framework for routine monitoring during the trial. This document will be based on UK MRC guidance ^72^ and indicate the extent (e.g. scope, detail, depth), timing, and reporting arrangements to be followed.

### Data sharing and access

A study-specific website will be established and have a linked web-based data repository, enabling data reuse and sharing. Access will be provided using web-based tools and applications. We will provide all necessary information including a description of the data repository system, and unique identifiers to allow it to be cited correctly. The study team will also make datasets available via the Open Science Framework and/or the Australian Research Data Common portal. Potential users can discover our data through these web-based resources and via open access publications and international conference presentations. Access to databases, survey tools, training materials and software tools developed by the study team will only be available for non-profit purposes. Consistent with MRC data sharing guidelines, our data management policy will outline the governance of access to research data. The TSC will have overall responsibility in deciding what categories of new users are able to access different types of information at different time points during and after completion of the trial, and will review applications by new users to undertake additional analyses using trial data.

The study team will have exclusive access to the data for 12 months after the primary data are published. Anonymised datasets that do not contain any participant identifiers or locator information will be made available for data sharing in order to maintain participant confidentiality.

External users of the data (i.e. outside of the research group) will be bound by data sharing agreements in accordance with MRC guidelines.

### Dissemination and publication policy

A dedicated study-specific website ( www.wantaim.org) will be used to disseminate progress updates to trial participants, stakeholders and partners. Updates may be provided as open access format reports and slide presentations; video updates from the field, including site visits and interviews with in-country policy makers; and interactive webinars (e.g. on trial rationale, progress and anticipated impact delivered by senior scientists).

Key stakeholders at national, provincial, district and health facility level will be provided with updates via periodic newsletters, technical reports and presentations provided by the in-country research team and investigators e.g. hospital grand round meetings; provincial health authority stakeholder meetings; meetings of the PNG Obstetrics and Gynaecology Society; National Department of Health Technical Advisory Committees and Working Groups. 

A National Policy Forum will be conducted on completion of the trial to enable senior health care managers, policy makers and development partners to engage with the research team in understanding the implications of our research findings for future public health policy. The forum will be preceded by early consultation with national and provincial policy-makers to establish locally meaningful benchmarks for feasibility and cost-effectiveness, so that any impact statements presented in the forum can address both international norms and local priorities for sustainability.

A Publication Policy will be developed and provided to the Trial Steering Committee for endorsement prior to the start of trial enrolment. Authorship will follow the guidelines of the International Committee of Medical Journal Editors. Scientific manuscripts arising from the trial will be submitted to peer-reviewed journals and published using the Creative Commons (CC-BY) open-access licence, wherever possible, and will be deposited in the PubMed Central (PMC) and European PMC repositories as soon as possible.

## Discussion

WANTAIM is the first randomised trial to evaluate the effectiveness, cost-effectiveness, acceptability and health system implementation requirements of point-of-care STI testing and treatment to improve birth outcomes in high-burden, low-income settings. If the intervention is proven to have an impact, and proves feasible, acceptable, and cost-effective for such settings, the trial will hasten access to these technologies, guide implementation, and could thereby improve maternal and neonatal health in high-burden, low-income settings worldwide.

### Trial status

Trial protocol version 0.3. Recruitment started July 2017. Estimated time to complete recruitment is 3 years.

### Trial registration

The trial is registered with the International Standard Randomised Controlled Trial Register ( *ISRCTN*) number: ISRCTN37134032.

## Data availability

### Underlying data

All data underlying the results are available as part of the article and no additional source data are required.

### Extended data

Open Science Framework: WANTAIM Trial Papua New Guinea. https://doi.org/10.17605/OSF.IO/N58PD ^60^.

The following extended data are available:

- WANTAIM_Community-Volunteer_Flipchart_PNG-Pidgin.pdf
- WANTAIM PISICF_English_v0.5 (23Feb2018).pdf (Participant information sheet and informed consent form)
- WANTAIM study brochure_English.pdf
- WANTAIM study brochure_PNG-Pidgin.pdf

### Reporting guidelines

Open Science Framework: SPIRIT checklist for “Point-of-care testing and treatment of sexually transmitted infections to improve birth outcomes in high-burden, low-income settings: Study protocol for a cluster randomized crossover trial (the WANTAIM Trial, Papua New Guinea)”. https://doi.org/10.17605/OSF.IO/N58PD ^60^.

Data are available under the terms of the Creative Commons Zero “No rights reserved” data waiver (CC0 1.0 Public domain dedication).
