# Supplementary material for: Point-of-care testing and treatment of sexually transmitted infections to improve birth outcomes in high-burden, low-income settings: Study protocol for a cluster randomized crossover trial (the WANTAIM Trial, Papua New Guinea)
Source: Wellcome Open Res. 2019 Nov 20;4:53. Originally published 2019 Mar 22. [Version 2] doi: 10.12688/wellcomeopenres.15173.2 (PMC6979472; doi:10.12688/wellcomeopenres.15173.2)
Supplement: Peer review history

# Peer review: Reviewer response for version 2

**DOI:** 10.21956/wellcomeopenres.17065.r37121

**Published:** 2020-01-13

**Authors:** Nicole Young

**Affiliations:** 1 Liverpool School of Tropical Medicine, Liverpool, UK

**23/01/2020 - This review has been updated from an Approved with Reservations to an Approved following a discussion between the authors and reviewers, which can be viewed beneath this peer review report at https://doi.org/10.21956/wellcomeopenres.17065.r37121.**

 Thank you very much your your response.

- I am happy to call this an effectiveness trial, but could you provide a discussion and scaled score of how 'explanatory' or 'pragmatic' the trial is across the 9 domains on the PRECIS-2 scale?

While trials often have the best intentions in mind, study conditions inherently will be different from the real-world implementation environments (although we try to minimize as much as possible!) eg. training provided by the study, additional research healthcare workers who will lesson the load of the routine clinic staff, supply of drugs/equipment that are not using the local logistic system etc. Having considered the differences would allow a deeper discussion of how to interpret trial results and what effect size to expect realistically in scale-up. This is especially true for resource strained locations where human resources are major bottlenecks in implementation.

- My comment on malaria is that there has been some evidence of SP having a protective effect on STIs (the exposure). Hence, it would be important to have data on those who received SP. 

I confirm that I have read this submission and believe that I have an appropriate level of expertise to confirm that it is of an acceptable scientific standard.

## References

[1] : The PRECIS-2 tool: designing trials that are fit for purpose. *BMJ*.2015;350: 10.1136/bmj.h2147 h2147 10.1136/bmj.h2147 25956159

# Response

**Published:** 2020-01-16

**Authors:** Andrew J. Vallely

**Affiliations:** PNGIMR, Papua New Guinea

Many thanks for these further comments and feedback.

**Comment #1:** I am happy to call this an effectiveness trial, but could you provide a discussion and scaled score of how 'explanatory' or 'pragmatic' the trial is across the 9 domains on the PRECIS-2 scale?

**Response #1:** Across the 9 domains of the PRECIS-2 tool, the trial scored 5 for domains 1,2,3,7 (‘Eligibility’, ‘Recruitment’, ‘Setting’ and ‘Follow-up’, respectively); scored 4 for domains 4,5,9 (‘Organization’, ‘Flexibility [delivery]’, and ‘Analysis’); and scored 3 for domain 6 (‘Flexibility [adherence]’). This indicates that the trial has a strong pragmatic focus, as described by Loudon K et al. (The PRECIS-2 tool: designing trials that are fit for purpose. BMJ 2015;350:h2147). We believe that it would be outside the scope of the current protocol paper to include this assessment in a further revised version of the manuscript, given that our trial is now underway and this assessment was not undertaken during the design phase (as recommended). We do however very much recognize the value and importance of this tool and will be using this approach during the design of future intervention trials in Papua New Guinea and other high-burden settings.

**Comment #2:** My comment on malaria is that there has been some evidence of SP having a protective effect on STIs (the exposure). Hence, it would be important to have data on those who received SP.

**Response #2:** We agree. As noted in our earlier comments, we are collecting data on possible confounders, including malaria (e.g. symptomatic and sub-clinical cases; exposure to SP for antenatal presumptive/preventative treatment) in the trial. We will describe in more detail how these factors will be taken into consideration in the trial Statistical Analysis Plan that we intend to publish separately.

# Response

**Published:** 2020-01-21

**Authors:** Nicole Yan

Thank you very much. Best of luck on your trial and I look forward to reading the results!

# Peer review: Reviewer response for version 1

**DOI:** 10.21956/wellcomeopenres.16555.r36067

**Published:** 2019-08-19

**Authors:** Nicole Young

**Affiliations:** 1 Liverpool School of Tropical Medicine, Liverpool, UK

Summary:

 This is a wonderful and important study to see if POC STI testing and immediate treatment would lead to better birth outcomes compared to standard of care using syndromic management. 

 It is a cluster-randomized cross over design that compares STI testing (chlamydia, gonorrhea, trich and BV) with POC diagnostics and syndromic management. The unit of randomization are health facilities and the primary outcome is a composite measure of the proportion of women who experience pre-term birth and/or low birth weight. The study also aims to assess cost-effectiveness and health system requirements of this testing strategy. 

**Abstract:**

 In the methods, please also describe the cross-over procedures as well.

**Introduction:**

1. Briefly describe how the timing of diagnosis of the STIs and treatment affects birth outcomes. For example, syphilis treatment is most effective in the early gestation period, and later treatment may not reverse damages to the fetus. Relate this to the importance of the timing of ANC visits and how this might affect the outcomes if early visits are not promoted in conjunction with the testing strategy. 
2. Under significance and potential impact of the trial: please provide a summary of the conflicting evidence, that gives more detail of the remaining questions that needs to be answered to better inform STI policy. Relate back to how this trial can shed new light on the existing evidence.
3. Because the study also aims to understand the 'acceptability and health system requirements of this strategy', please also review the literature on implementation challenges of POC diagnostics. Currently there is very little description of health system requirements and would warrant a paragraph or so. Who will be the intended healthcare workers to carry out this testing? Would it be the laboratory staff or the ANC nurses/clinicians/or person who does the HIV testing if different from the ANC nurse? I suggest using an implementation framework to do this. See: Tanahashi T. Health service coverage and its evaluation. Bull World Health Organ ^1^.

For POC challenges, see:

- Young N, Taegtmeyer M, Aol G, Bigogo GM, Phillips-Howard PA, et al. (2018) Integrated point-of-care testing (POCT) of HIV, syphilis, malaria and anaemia in antenatal clinics in western Kenya: A longitudinal implementation study ^2^.
- Young N, et al. Integrated point-of-care testing (POCT) for HIV, syphilis, malaria and anaemia at antenatal facilities in western Kenya: a qualitative study exploring end-users’ perspectives of appropriateness, acceptability and feasibility ^3^.

**Protocol:**

Trial aim and objectives

- As this is a trial, the 'effectiveness' (performance of the intervention in real-world conditions) is not being measured.  It may be more appropriate to say it is measuring the 'efficacy' (performance in controlled conditions) of the intervention. See table 1 of the below reference:

See: Singal AG, Higgins PD, Waljee AK. A primer on effectiveness and efficacy trials ^4^.

Procedures

 As SP is given for malaria prophylaxis, please explore and discuss SP's protective effect on STIs (in the background). It would be important to document and ensure that the proportion of women given SP is balanced between the arms or it may need to be adjusted for in the end (under the statistical analysis section). Coverage of facility level delivery of SP has been shown to be low and this may be due to a multitude of implementation/health system/patient-provider factors that may not have been accounted for in the randomization process. (see: Hill J, Dellicour S, Bruce J, Ouma P, Smedley J, et al. (2013) Effectiveness of Antenatal Clinics to Deliver Intermittent Preventive Treatment and Insecticide Treated Nets for the Control of Malaria in Pregnancy in Kenya ^5^.

 For SP's protective effect on STIs see: 

- Chico RM, Moss WJ. Prevention of malaria in pregnancy: a fork in the road? ^6^. 
- Chico RM, Chaponda EB, Ariti C, Chandramohan D. Sulfadoxine-Pyrimethamine Exhibits Dose-Response Protection Against Adverse Birth Outcomes Related to Malaria and Sexually Transmitted and Reproductive Tract Infections. Clinical infectious diseases : an official publication of the Infectious Diseases ^7^. 
- Gutman J, Slutsker L. Intermittent Preventive Treatment with Sulfadoxine-Pyrimethamine: More Than Just an Antimalarial? The American journal of tropical medicine and hygiene ^8^.
- Capan M, Mombo-Ngoma G, Makristathis A, Ramharter M. Anti-bacterial activity of intermittent preventive treatment of malaria in pregnancy: comparative in vitro study of sulphadoxine-pyrimethamine, mefloquine, and azithromycin ^9^.

Please clearly describe the number of clinical research staff that would be given to the facilities and their roles. Do these clinical research staff have medical/nursing training and would help with routine clinic services? Who will be conducting the routine antenatal clinic procedures and the study-specific procedures? It seems that the study-specific procedures would require the skills of a nurse and this nurse will likely then be also conducting the routine antenatal procedures. 

 This will have implications for human resources if the intervention were to be handed over to the routine clinic staff. Because the study also aims to understand the health system requirements of this POC strategy , it would be important to interpret the trial findings (especially secondary outcome #5) with having this additional research staff at the facilities in mind. 

 How will HIV and syphilis be tested? Is syphilis done using rapid tests or laboratory based testing (VDRL/RPR)? 

Collection of health system implementation data and acceptability data

 For the evaluation of health system data, it is important to be aware of the conceptual distinction among different implementation outcomes (eg. acceptability, feasibility). This will allow better understanding of implementation processes and comparison of any strategies. Please summarize the implementation outcomes this study will evaluate and how each of the data collection procedures will address/measure the outcome. Suggested table may consist of 4 columns: col 1- implementation outcome; col 2- construct definition;  col 3- metric; col 4 - data collection source for that metric

 see: Proctor E, Silmere H, Raghavan R, et al. Outcomes for implementation research: conceptual distinctions, measurement challenges, and research agenda ^10^. 

**Trial design and setting**

1. Please provide more details of the study settings - types of health facilities, number of health facilities in the catchment area, facility volume, healthcare worker mix for MCH
2. Please provide a rationale for cross-over design 
3. How long will each of the phases take?

I confirm that I have read this submission and believe that I have an appropriate level of expertise to confirm that it is of an acceptable scientific standard, however I have significant reservations, as outlined above.

## References

[1] : Health service coverage and its evaluation. *Bull World Health Organ*.1978;56(2) :295-303 96953PMC2395571

[2] : Integrated point-of-care testing (POCT) of HIV, syphilis, malaria and anaemia in antenatal clinics in western Kenya: A longitudinal implementation study. *PLoS One*.2018;13(7) : 10.1371/journal.pone.0198784 e0198784 10.1371/journal.pone.0198784 30028852PMC6054376

[3] : Integrated point-of-care testing (POCT) for HIV, syphilis, malaria and anaemia at antenatal facilities in western Kenya: a qualitative study exploring end-users' perspectives of appropriateness, acceptability and feasibility. *BMC Health Serv Res*.2019;19(1) : 10.1186/s12913-018-3844-9 74 10.1186/s12913-018-3844-9 30691447PMC6348645

[4] : A primer on effectiveness and efficacy trials. *Clin Transl Gastroenterol*.2014;5: 10.1038/ctg.2013.13 e45 10.1038/ctg.2013.13 24384867PMC3912314

[5] : Effectiveness of antenatal clinics to deliver intermittent preventive treatment and insecticide treated nets for the control of malaria in pregnancy in Kenya. *PLoS One*.2013;8(6) : 10.1371/journal.pone.0064913 e64913 10.1371/journal.pone.0064913 23798997PMC3683044

[6] : Prevention of malaria in pregnancy: a fork in the road?. *The Lancet*.2015;386(10012) : 10.1016/S0140-6736(15)00325-6 2454-2456 10.1016/S0140-6736(15)00325-6 26429701

[7] : Sulfadoxine-Pyrimethamine Exhibits Dose-Response Protection Against Adverse Birth Outcomes Related to Malaria and Sexually Transmitted and Reproductive Tract Infections. *Clinical Infectious Diseases*.2017;64(8) : 10.1093/cid/cix026 1043-1051 10.1093/cid/cix026 PMC539994028329383

[8] : Intermittent Preventive Treatment with Sulfadoxine–Pyrimethamine: More Than Just an Antimalarial?. *The American Journal of Tropical Medicine and Hygiene*.2017;96(1) : 10.4269/ajtmh.16-0888 9-10 10.4269/ajtmh.16-0888 PMC523971627994102

[9] : Anti-bacterial activity of intermittent preventive treatment of malaria in pregnancy: comparative in vitro study of sulphadoxine-pyrimethamine, mefloquine, and azithromycin. *Malar J*.2010;9: 10.1186/1475-2875-9-303 303 10.1186/1475-2875-9-303 21029476PMC2984572

[10] : Outcomes for implementation research: conceptual distinctions, measurement challenges, and research agenda. *Adm Policy Ment Health*.2011;38(2) : 10.1007/s10488-010-0319-7 65-76 10.1007/s10488-010-0319-7 20957426PMC3068522

# Response

**Published:** 2020-01-07

**Authors:** Andrew J. Vallely

**Affiliations:** PNGIMR, Papua New Guinea

Many thanks for these valuable comments, which we have responded to in the revised version of the manuscript. A brief summary of our responses to specific comments is provided below for ease of reference:

**Abstract:** it was not possible to include additional information on cross-over procedures due to word limit restrictions.

**Introduction:** a brief summary of conflicting international evidence has now been added; information on the timing of STI diagnosis and treatment has also been added to the ‘Trial design’ section. We will be presenting the health systems component of the trial in detail in a separate protocol manuscript to be submitted in the first quarter of 2020.

**Trial objectives:** we consider this an effectiveness trial because it is being conducted under real-world conditions in routine clinical settings. This definition has been endorsed by our Trial Steering Committee and Data & Safety Monitoring Board.

**Trial design:** information on the rationale for selecting a crossover design, selection of trial clusters and duration of trial phases, are now included in the revised manuscript.

**Procedures:** field team composition, roles and responsibilities have been added. We are collecting data on potential confounders, including malaria, and will describe in more detail how these factors will be taken into consideration in the trial Statistical Analysis Plan, which we will plan to publish separately in the first quarter of 2020.

# Peer review: Reviewer response for version 1

**DOI:** 10.21956/wellcomeopenres.16555.r35670

**Published:** 2019-06-18

**Authors:** Matthew Hamill

**Affiliations:** 1 School of Medicine, Johns Hopkins University, Baltimore, MD, USA

This is an incredibly important area and one worthy of such high quality and thoughtful research as set out in the study protocol by Vallely et al. It is very exciting and a pleasure to read. The protocol outlines a planned cluster randomized cross over control trial (WANTAIM) which will take place in antenatal and postnatal care in Papua new Guinea. The study is large (n=4000) and designed is to compare standard-of-care management of sexually transmitted infection (STI) symptoms with those informed by point of care tests for 4 sexually transmitted or associated infections in pregnant women.  

 The primary outcome is a composite of preterm birth (<37 weeks) and/or low birth weight (<2500 g) within 72 hours of birth. There are several interesting secondary outcomes including a sub-study of 2000 infants to test for neonatal chlamydial or gonococcal eye infection.

 There are a few areas that the authors might like to consider.

1. The lack of blinding and informational material given to the control groups may result in contamination. Women, their partners and providers may be more aware of STI as a result of the trial meaning that women may be more likely to complain of symptoms and their providers more likely to enquire into genital symptoms and, as a result, more likely to provide syndromic management. Such an effect may reduce the observed difference in favor of the null.
2. The study visits are potentially very close together e.g. visits 2 and 3 may be well less than a month apart. This introduces the potential for false positive STI tests where the infection has been treated but non-viable DNA is detected giving a positive test. It will be important to ensure that women who test positive and who are treated are not tested too soon after the initial positive result.
3. Has the role of group B strep been discussed and it is not being tested for. There is a POCT for GBS and its presence/absence may skew results.
4. A single HIV test at enrolment visit will miss those with exposure later in pregnancy that may be facilitated by STI. Incident HIV (using DBS) could be an interesting secondary outcome. In the same vein it was not clear if HIV-infected women will continue in the study or be excluded based on the comment: 'Women who test positive for HIV infection will be referred for specialist review and initiation of antiretroviral therapy'.
5. My main methodological concern is that STI in control group will be tested for using lab-based NAAT on **urine** compared to **vaginal swab **in intervention group. The Vaginal swab tends to perform better than urine in women so potentially biasing results towards intervention. Regardless STI positivity will be determined using different sample types so it will be important to test them against each other.
6. What test is being used to test for BV in urine? This is the implication for testing in the control group.
7. The NPA on the babies for CT may be too soon (as per point 2) rising the potential for false positive results. I am not aware of time to negativity in NPA after CT treatment. 

Finally, could the authors add a reference after comparing GeneXpert and lab-based NAATs.

 This is a wonderful study and I am very much looking forward to the results which may argue for a major shift in policy in resource limited settings.

I confirm that I have read this submission and believe that I have an appropriate level of expertise to confirm that it is of an acceptable scientific standard.

# Response

**Published:** 2020-01-07

**Authors:** Andrew J. Vallely

**Affiliations:** PNGIMR, Papua New Guinea

Many thanks for these valuable comments, which we have addressed in the revised version of the manuscript. A brief summary is also provided below for ease of reference.

**Group B strep (GBS):** residual urine and genital specimens are being retained at all visits and will enable us to investigate potential confounders (e.g. GBS, *M. genitalium*) following completion of the trial.

**HIV status:** the trial does not have sufficient power to investigate incident HIV infections (antenatal HIV prevalence is around 0.6% in PNG); women found to be HIV positive are referred for specialist review and treatment (available only at provincial-level) after which they may continue antenatal follow-up as part of the trial or continue provincial-level services.

**Neonatal specimens:** are collected at multiple time points to evaluate the impact of the intervention on both exposure and infection.

**Vaginal vs urine specimens: **Xpert STI test performance is comparable using urine or vaginal specimens (e.g. sensitivity 98%, specificity 99% for detection of chlamydia using either specimen type: https://www.ncbi.nlm.nih.gov/pubmed/23467600); BV testing is not being conducted in the control arm **.**

**References comparing Xpert and lab based NAATs:** have now been added.
